# Zygotic-splitting after in vitro fertilization and prenatal parenthood testing after suspected embryo mix-up – a case report

**DOI:** 10.1007/s00414-024-03245-9

**Published:** 2024-05-02

**Authors:** Iris Schulz, Janine Schulte, Dorothea Wand Dipl-Med

**Affiliations:** 1 Institute of Forensic Medicine, Health Department, University of Basel, Basel-Stadt Pestalozzistrasse 22, Basel, CH-4056 Switzerland; 2 University Hospital Basel, Institute of Medical Genetics and Pathology, Schönbeinstrasse 40, Basel, 4031 Switzerland

**Keywords:** Late twinning, Embryonic mix-up, Short tandem repeat (STR) analysis, Relationship testing, Assisted reproductive technology (ART), *In vitro* fertilization (IVF)

## Abstract

After in vitro fertilization with a single embryo, the parents learned about being pregnant with twins in the 10th week with various indications that an embryonic mix-up could have taken place. The affected couple thus expressed the urgent desire for a clarification of parenthood considering an abortion. However, the prenatal test results would not have been available until the 14/15th week of pregnancy. Legally, then, severe physical or mental distress of the pregnant woman must be claimed by physicians to justify an abortion after the twelfth week. However, a lack of genetic relatedness could lead to serious psychological distress for the parents, making a pregnancy termination possible even after the twelfth week, which is discussed in this case study alongside the interdisciplinary team’s ethical, legal, and medical considerations.

For the invasive relationship testing, cultivated chorionic villi samples (CVS) from both unborn and saliva samples from the putative parents were genetically analyzed using classical short tandem repeats (STR) analysis. The perfect match of both CVS profiles suggested the occurrence of an unusual late twin shaft, for which, fortunately, parenthood could be confirmed. To our knowledge, this is the first report on a prenatal investigation of a suspected embryo mix-up after assisted reproductive technology (ART), in which parenthood should be fixed. We want to draw attention to this unthinkable scenario, which may increase in the future with ART-induced rising multiple pregnancies.

**Supplementary Information:**

The online version contains supplementary material available at 10.1007/s00414-024-03245-9.

## Introduction

Statistical reports from the European Society of Human Reproduction and Embryology show a significant trend in industrialized countries in the number of recorded twin and multiple pregnancies from the late 90s to the present [1], as also recently published by Monden et al. [2]. One reason for this increase is the intensified use of medically assisted reproduction (MAR) in the last decades [3]. In addition to simple ovarian stimulation, MAR also includes assisted reproductive technology (ART) with in vitro treatment of male and female germ cells, i.e., in vitro fertilization (IVF) and intracytoplasmic sperm injection (ICSI). Another reason for spontaneous multiple pregnancies is probably the advanced average age of childbearing women (> 35 years), which is known to be associated with multiplicity in offspring [4, 5]. Beemsterboer et al. explained the paradox of declining fertility and higher twinning of aging women by an increased tendency towards multiple follicular developments. This is, in turn, associated with rising Follicle-stimulating hormone concentrations with advancing maternal age. However, in some countries, the incidence of multiple pregnancies after ART has decreased again, which is likely related to the ‘Reproductive Medicine Act’ revision that limits the transfer to a single embryo to minimize the health risks to the unborn [6].

A closer look at human conception and resulting embryonic development helps to understand the different classifications of dizygotic (DZ) and monozygotic (MZ) twins. The fertilized oocyte, the zygote, migrates to the uterus and forms a cluster of cells that divide and grow to a so-called blastocyst. Reaching the uterus, the blastocyst implants into the endometrium [7]. During IVF, healthcare providers can do preimplantation genetic testing (PGT) and take cells at the blastula stage to check which embryos are healthy and have the greatest chance of successful implanting once transferred to the uterus. Together, the outer cells of the blastocyst and the uterine inner lining will form the future placenta for protection, nourishment, and oxygen supply [8]. If the blastula cells implant successfully, they evolve into an embryo. After 10 to 12 weeks of pregnancy, the embryo moves into the final stage of development, a fetus. Here, the individual grows in a fluid-filled membranous sac, a thin transparent pair of membranes [7]. The outer, thicker layer is the chorion, a part of the placenta. The chorion contains a second and thinner layer on the inside, the amnion. The latter is the fetal part of the placenta, which encloses the amniotic cavity, holding the embryo within an amniotic fluid [7, 8].

In most cases, twins arise from two zygotes (dizygotic), with an average incidence of 13 per 1000 live births worldwide [9], ranging from as low as 2 per 1000 in Asia and South America to 40 per 1000 in African countries [2, 4]. Fraternal twins form when two separate eggs are released and fertilized by two sperm, thus with different genetic contributions like siblings of the same or different sex and with separate placentas and membranes (dichorionic diamniotic) [10]. DZ twins are common with fertility treatments that cause multiple eggs to be released or more than one embryo to be transferred into the uterus. In contrast, monozygotic twins are a rarer phenomenon in natural conceptions at 3.5 to 4 per 1000 births but twice as common in assisted births [11, 12]. MZ twins result from fertilizing one egg with one sperm to form one zygote, in which the blastocyst then splits prior to its attachment to the uterus and develops into two embryos. While the split product of a single fertilized ovum results in (nearly) genetically identical offspring and consequently the same gender, MZ twins can undergo diverse (epi-)genetic changes during pre- and postnatal development. With these alterations, MZ twins are not entirely genetically identical, thus allowing for forensic discrimination [13, 14].

**Fig. 1:**
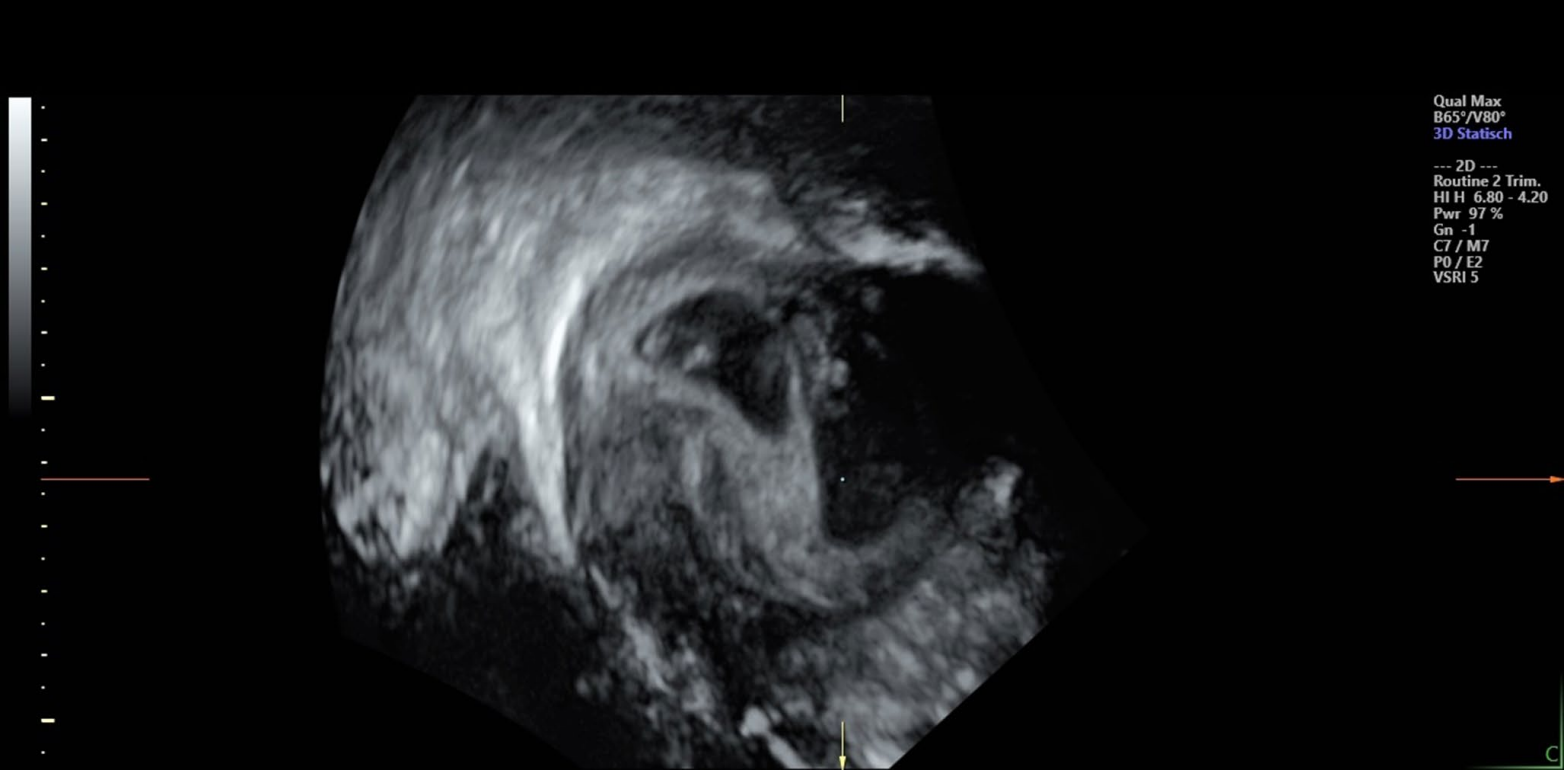
Ultrasound images from the 10th week of a dichorionic diamniotic twin pregnancy, where the twins are separated by a thick layer of fused chorionic membranes. Patient permission has been granted

Typically, three clinical classifications of monozygotic twin divisions are described, using their placental membranes to distinguish zygosity: (1) The splitting of the zygote within the first two or three days after fertilization results in a dichorionic diamniotic (DCDA) pregnancy, where each embryo has its chorion and amniotic sac [15]. (2) If MZ separation occurs between four and six days postfertilization, in 70–75%, the chorionic cavity carries both twins, isolated by the amniotic membranes (monochorionic diamniotic, MCDA). However, 25–30% of these MZ twins also show separate placentas and membranes. (3) Finally, monochorionic monoamniotic (MCMA) twins share a placenta, and as they have no amniotic membrane between them, they are at risk of vascular compromise because of the twisting of their umbilical cords around each other. MCMA twins develop only in 1–2% of monozygotic twinning cases after the sixth day after fertilization when hatching and implantation in the uterus begin [10]. Thus, the general classification of monozygotic twin pregnancies is based on the time and mode of division of the fertilized ovum, leading to a different number of placentas and amniotic sacs (Fig. 1).

Knowing the different timelines and kinds of twin formation was of great importance for the presented case - a gemini pregnancy after transferring a single embryo in the ICSI cycle. For twin differentiation in DCDA, MCDA, or MCMA, diagnosis is regularly conducted by ultrasound, which is well-established in obstetric care [16, 17]. Here, ultrasonography at week ten revealed that the twins were DCDA, i.e., each fetus had its placenta and amniotic sac. According to the aforementioned criteria, this twinning type would have been expected for blastocyst cleavage within the first three days after fertilization. However, the responsible foreign clinic transfers the zygote exclusively at a late blastula stage, which – if twinning had occurred – an MCDA or MCMA pregnancy would have been expected. In addition to the clinically unexpected twin type, other factors increased uncertainty about correct embryonic implantation. For instance, information about the number of the patient’s remaining embryos was contradictory. Further, another woman should have had multiple embryos transferred on the same day of the patient’s implanting treatment. Moreover, the clinic abroad refused to provide additional requested documentation about the implantation. Therefore, the couple suspected that the planned transfer of one fertilized oocyte was a mix-up with the other clinic case, with multiple embryos to be implanted. Although the couple wished to have a child and, in principle, would have adopted twins, the doubts about whether the embryos were their biological children caused an emerging desire to terminate the pregnancy unless genetic confirmation of their parenthood was proven. While it could be contradictorily discussed to abort a child after an artificial treatment, it must be stated that the possible event of an embryonic mix-up clearly is not a free decision of either of the couples involved and such potential error should be clarified with respect to the genetic confirmation of parenthood.

Prenatal sampling for relationship clarification is controversially discussed, with, on the one hand, associated risks for the unborn (i.e., injuries, deformation, miscarriage) [16, 18, 19], but also for the mother (i.e., maternal mortality [20, 21]). On the other hand, in many advanced societies, women have the right to determine their reproductive health. Currently, this human right is being heavily debated in light of the latest U.S. Supreme Court overturning the federal standard protecting the right to abortion [22]. For both non-invasive and invasive examinations, country-specific legislation ranges from illegal with specified medically- or crime-related exceptions, as in Germany [23], to legitimate, as in Switzerland [24]. In *in-vitro* fertilization, parenthood should generally not be in question with known female and male gamete donors. However, in insemination, gamete mix-ups might occur, with unimaginable scenarios for the affected couples, as reported last year [25].

To our knowledge, this case report is the first to present a short tandem repeat (STR) based prenatal parenthood clarification after a suspected embryo mix-up in ART-induced fertilization, in which the relationship should be undisputed. However, this inexplicable scenario could rise as the number of MAR-related multiple pregnancies increases. Therefore, we aim to draw attention to this unusual prenatal kinship testing and highlight ethical, legal, and medical considerations addressed during case management processing.

## Methods

### Case report

In her tenth week of pregnancy after ART treatment of a single embryo, the patient learned of being pregnant with twins. Both parents were essentially healthy.

### Ethical and prenatal consultation

Before analysis, the interdisciplinary counseling team discussed the case-related legal, ethical, and clinical aspects and concerns of prenatal parentage testing and possible abortion in the case of unrelated offspring. The couple received detailed and educational counseling and support services clarifying parentage and related consequences. The parents’ written declaration of consent was obtained for the genetic analysis to be performed. Information on the couple’s family history, ethnic background, past genetic, obstetrical, medical, and surgical history was also evaluated.

### DNA sampling, extraction and STR genotyping

For twin testing, a medical professional from the Institute of Medical Genetics and Pathology, University Hospital Basel, took CVS (each ~ 25 mg) from the two placentae being in close proximity to each other. To avoid getting chorion material from the same embryo twice, the sampling was performed as far apart from both chorion regions as possible. After CVS cultivation, DNA was extracted from both cell cultures by the Institute of Medical Genetics and Pathology and sent to the Institute of Forensic Medicine, University Basel, for downstream molecular genetic analysis. In mutual agreement, cheek mucous membrane samples were taken from the putative parents by the Institute of Forensic Medicine. DNA extraction was performed for all samples in duplicates using SwabSolution™ Kit according to the manufacturer’s protocol (Promega Corporation, Madison, USA) with an elution volume of 50 µl. As for standard relationship testing, the samples were not quantified.

In the Institute of Forensic Medicine, autosomal STRs were genotyped using the multiplex PCR kits PowerPlex® ESI 17 Fast System and PowerPlex® Fusion 6 C (both Promega), following the manufacturer’s recommendations with 0.5 ng extracted DNA and a total PCR volume of 25 µl. Amplified PCR products were separated by capillary electrophoresis on an ABI PRISM 3500xL genetic analyzer (Applied Biosystems, Foster City, USA) and analyzed with GeneMapper™ ID-X v.1.5 Software (Applied Biosystems) using validated threshold of 50 resonance fluorescent units.

### Statistical calculations

For parenthood testing, likelihood ratios (LR) were used. LR is a measure of the power of proof regarding the following alternate hypotheses: H0 (the putative mother and the putative father are the biological parents of the unborn twins) versus H1 (two unknown and unrelated persons to the tested adults are the biological parents of the unborn twins). The biostatistical calculation was carried out with the help of the VAT statistics program by Prof. Dr Max Baur, the University of Bonn, based on the European population data from Caucasian descent. Statistical analysis for the twin’s monozygosity of the unborn was unnecessary and thus not performed [13, 26, 27].

## Results and discussion

**Fig. 2:**
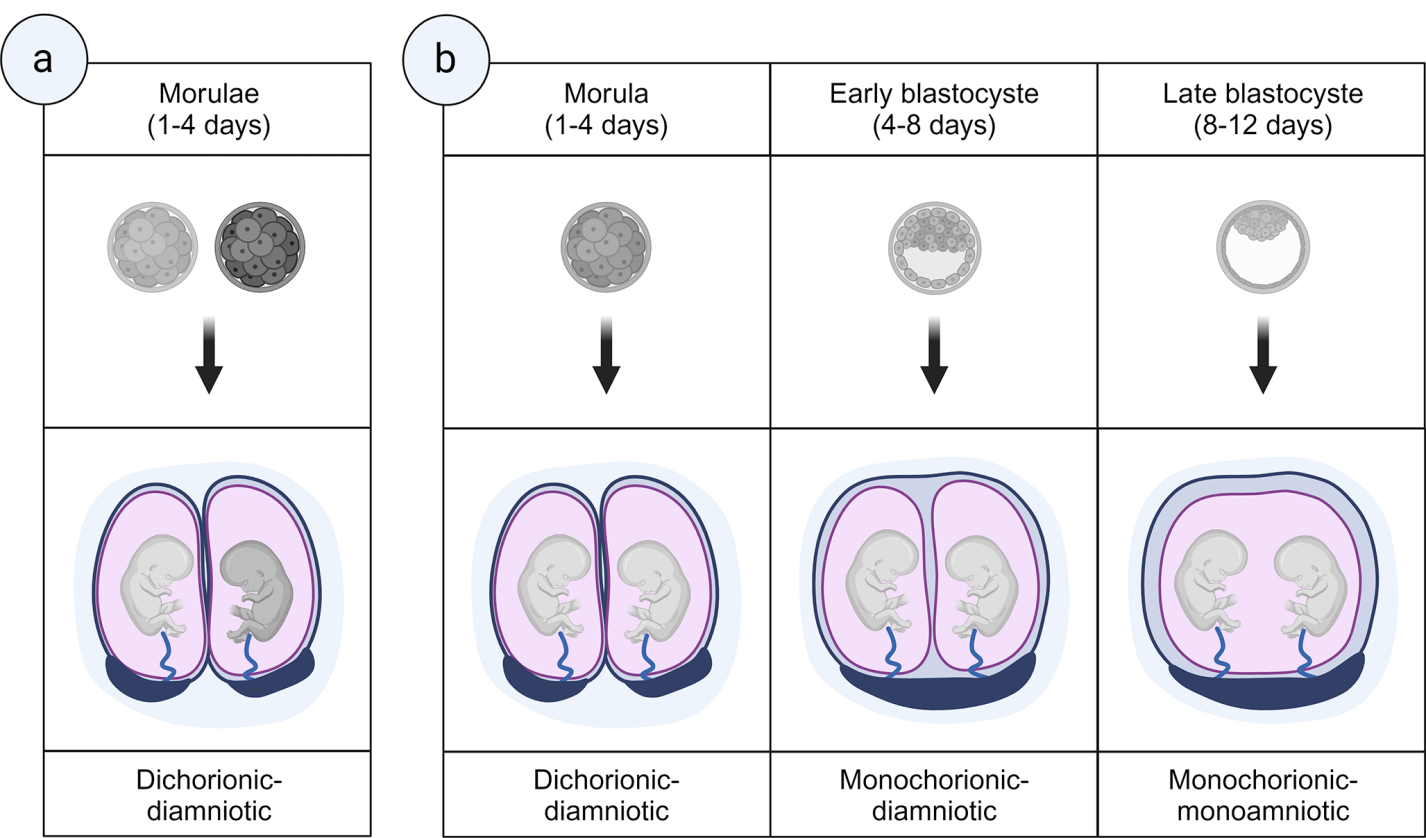
Dizygotic (**a**) and monozygotic twinning and placenta (**b**). Dichorionic-diamniotic (occurrence of 30–34% or 32–35%) and monochorionic-diamniotic (occurrence of < 1% or 21–30%) can have one or two placentas, with cleavages reported at morula or early blastula stages. Monoamniotic monochorial twins always share one placenta (occurrence of 1%) with a late blastula cleavage. Conjoined twinning occurs when the zygote cleaves after 13 days, resulting in embryos that are abnormally fused (not shown). Figure created with BioRender.com following [46]

Ultrasonography at twelfth weeks’ gestation revealed a DCDA pregnancy (Fig. 2), i.e., two fetuses with one chorion each. The histopathological assessment determined dichorionic placenta weights of 306 g and 437 g, respectively. The examinations during pregnancy, especially ultrasound, were all unremarkable. In the 38th week of pregnancy, two healthy children with regular birth masses were born.

Standard STR profiling resulted in a perfect match between the DNA profiles of both CVS samples. With clinical certainty that the prenatal material came from two different chorions and thus embryos, the matched DNA profiles provided evidence of an identical twin split that unexpectedly occurred at the late blastula stage. For the identical prenatal DNA profiles, no exclusion of parenthood was found in 23 STRs. The combined statistical calculation yielded an index of 7.6415*10^20^ for the assumed parenthood and, thus, confirmed the MZ twins as the biological offspring of the parents in question.

The concerned couple had explicitly opted in advance for the implantation of a single embryo. After the gemini pregnancy was known, the reproductive fertility center abroad had provided written assurances that only one embryo had been transferred but otherwise failed to respond to any of the parents’ further inquiries and concerns about the patient’s remaining egg cells and the parallel treatment of another woman with two implants at the clinic. Thus, for the expectant patient, the insemination of foreign embryos seemed more likely than a possible late twinning of the blastula. Suspecting an embryonic mix-up, the parents urged clarifying their relationship to the unborn if possible or, if not, a pregnancy termination. By law, severe physical or mental distress of the expectant mother must be claimed to justify a pregnancy termination after the twelfth week. Therefore, an interdisciplinary counseling team was consulted in the 11th week of pregnancy, consisting of legal, medical, and ethical professionals. Their responsibility was to provide advice and support in the decision-making process of whether to continue or terminate the pregnancy, taking into account the country-specific legislation, medical risks, and ethical considerations. Further, the results on parenthood could not have been expected before the 14th /15th week of pregnancy due to the necessity to cultivate the CVS cells prior to the genetic analysis, which complicated the legal (i.e., later than week 12) and ethical terms (i.e., fully developed fetus). To this end, the presumption of an embryonic mix-up, along with the related doubts and insecurities, caused significant physical and psychological stress for the couple concerned, making a prenatal clarification of parenthood indispensable. Thus, surgical abortion would have been legally permissible, even after the 12th week of pregnancy [20].

To make an informed decision, the expectant parents must be precisely informed about the possibilities and risks of prenatal procedures. In addition, the interdisciplinary council provided and discussed the following alternatives, among others:

1. An abortion within the timeline up to the 12th week of pregnancy without genetic testing;
2. A genetic test and carrying the pregnancy to term, with the prospect of exchanging the children if they are not related; or,
3. A genetic test with the prospect of a legal termination after the 12th week of pregnancy if unrelated.

Concerning the first option, an abortion can be legally performed within the first twelve weeks of pregnancy [20]. However, due to the physician’s duty of care, the patient should be advised against abortion based on an unconfirmed suspicion of embryo mix-up during in vitro fertilization, particularly in view of their desire to have a child. The second option, carrying the pregnancy to term, followed by the subsequent release of the children for adoption between the concerned couples, was also considered. Child exchange followed by adoption would require that the other woman involved was pregnant with the patient’s embryo, would be informed, carry the couple’s child to term, and agree to an exchange of offspring. Option 2 seemed to be very questionable to be feasible in practice, as it would mainly depend on the involuntary willingness of surrogacies for both women (coping with mental and psychological stress to carry out a foreign child), provided excellent cooperation with the IVF center abroad, in addition to data protection issues. Therefore, the third option, the prenatal test for relatedness and possible post-twelfth-week termination, was the remaining choice for the couple, also supported by the counseling board.

For prenatal analysis, fetal material can be obtained invasively from the 10th week of gestation by chorionic villus biopsy [16], from the 15th week by amniocentesis [16], and from the 18-20th week by cordocentesis [28]. Noninvasively, fetal cell-free (cf) DNA can be examined, which is present in the maternal bloodstream from the 9th week onwards [29]. This ‘liquid biopsy’ allows clarification of the paternity of the unborn child without any severe or fatal risks. From the ninth week of pregnancy, the developing fetus has produced so many cells that numerous cf DNA fragments circulate in the mother’s bloodstream [30]. However, in twins, the method has shown an increase in the total fetal fraction in maternal material but with decreased fractions per fetus [31, 32], with possibly indeterminate or uninterpretable results, which have also been observed for non-strain single prenatal tests [30].

Regarding multiple pregnancies, likewise, a paucity of data exists for invasive procedures [33], but these biopsies are common and well-established in prenatal diagnosis of singletons [16, 17]. For cordocentesis, one option is to introduce a needle at the placental cord insertion site to obtain material from the fetus [28]. In amniocenteses, a needle is inserted through the mother’s abdominal wall to remove some amniotic fluid, in which fetal cells are present. However, both surgeries could not be considered due to the late stage of pregnancy (second trimester), leaving CVS as the remaining and recommended procedure. Despite being commonly used, this first-trimester biopsy is still associated with the small but existent procedural risk of miscarriage reported in a range of 0.03–0.3% [29, 34]. During this surgical procedure, a small piece of the placenta is removed, from which the embryo forms half of the tissue that can be further investigated for its genetic origin.

In the presented parenthood clarification, another question was raised, namely whether the chorionic villi of both embryos could and should be collected and if – due to close proximity – the same individual could be sampled twice by chance. As MZ twins share the genetic origin, their DNA profiles are expected to be identical [35]. Hence, STR analysis would not reveal whether CVS of one or both MZ fetuses were taken. By DNA sequence analysis, however, existing genome differences, e.g., rare *de novo* mutations between germlines [26], could be identified and potentially used for MZ twin differentiation [13, 27]. Nevertheless, the probability of genetic relatedness to only one fetus was considered very low, which could have originated by spontaneous conception before ART or due to contamination with the zygote of the other parents. After consultation with the medical physician for CVS sampling, differentiation of the unborn twins, although not easy, was possible without reasonable doubt. With an error considered minor, the chorionic villi provided were assumed to derive from two individuals with proven parentage for the twin pair.

Prior to the discussion of prenatal sampling and genetic analysis, another less obvious challenge and a potential source of mistake was also addressed: prenatal twin determination with potential pitfalls in the accuracy of chorioamnionicity [36]. Errors in twin sonographic interpretation can happen, which in the presented case could have falsely led to the DCDA designation, which reinforced the doubt of correct embryonic implantation as one factor. For example, Lu et al. described twins at week 12 initially believed to be DCDA based on a thick “intertwin membrane”. However, the second ultrasound revealed that this “membrane” was an intrauterine septum. Thus, the true intertwin membrane had only two layers of amnion, revealing monozygotic twinning. Another error could be caused by a rare occurrence of a bilobate placenta in monochorionic twins (3% of monozygotic twinning [37]). The phenomenon describes two nearly equal-sized placental lobes connected by chorion leave, mimicking dichorionicity. Taking into account a possible misinterpretation of the DCDA assignment, an MDMA or MDMC twin type would have been more likely for the affected couple with respect to the blastula split time point, which would have theoretically made the prenatal test unnecessary.

For late twinning of the blastula, it has been suggested that the number of incidents increases due to MAR. For example, ovulation induction [38], patient age [39, 40], or duration of cultivation [41–44] were shown to have a positive impact on the occurrence of multiplicity pregnancies. Others proposed that the high incidence of monozygocity in infertility-clinic patients was conditioned by hereditary factors, enhanced by excellent ovarian function [45]. However, during preliminary medical clarification, no monozygotic or dizygotic twinning in the parents’ families was observed within the last four generations. Thus, late twinning at the blastula stage was the fortunate explanation for the observed twin pregnancy reported here.

## Conclusion

Under state-specific legislation, expectant couples have certain rights, such as the right to a legal abortion within the first 12 weeks of pregnancy or later if a counseling board determines that the patient is suffering severe emotional and/or physical distress. The advisory board offers the opportunity to provide accurate and transparent information about the existing options to assist the decision-making process of carrying the pregnancy to term or terminating it. Here, the interdisciplinary professional exchange also supported the involvement of the forensic genetic team. Knowing that the parents had opted to terminate the pregnancy if prenatal testing was not available supported the decision to perform the investigations. Nevertheless, the knowledge that a non-relationship between the offspring and the couple would inevitably lead to an abortion was difficult to bear, despite an understanding of the woman’s autonomy and the otherwise involuntary surrogacy.

While in the presented case the relationship of the unborn was fortunately proven, such confusion of embryos might occur during technically assisted procedures, with unimaginable consequences for the persons concerned. To minimize the risk of embryonic mix-up, multiple checks are performed throughout the artificial insemination process, including different medical professionals independently confirming the correct identity of the material (i.e., sperm, oocyte) and a strict chain of custody, as is standard practice in medical and forensic investigations. Unfortunately, but not unexpectedly, the authors’ attempt to obtain any information on possible known failure rates remained unanswered. Even though it is assumed to be rare, the possibility of donor germ mix-up cannot be ruled out, as humans can err. With this unusual case report, we aimed to draw attention to this unthinkable scenario, which might increase in the future with ART-induced rising multiple pregnancies, to highlight the influence and necessity of interdisciplinary exchange, and to plead for a transparent handling of failure rates of the associated technical processes.

### Electronic supplementary material

Below is the link to the electronic supplementary material.

Supplementary Material 1

## Data Availability

Data sharing is not applicable to this article as no datasets were generated or analyzed during the current case report.
