# Supplementary figures and images for: Zygotic-splitting after in vitro fertilization and prenatal parenthood testing after suspected embryo mix-up – a case report
Source: Int J Legal Med. 2024 May 2;138(5):2057–64. doi: 10.1007/s00414-024-03245-9 (PMC11306302; doi:10.1007/s00414-024-03245-9)

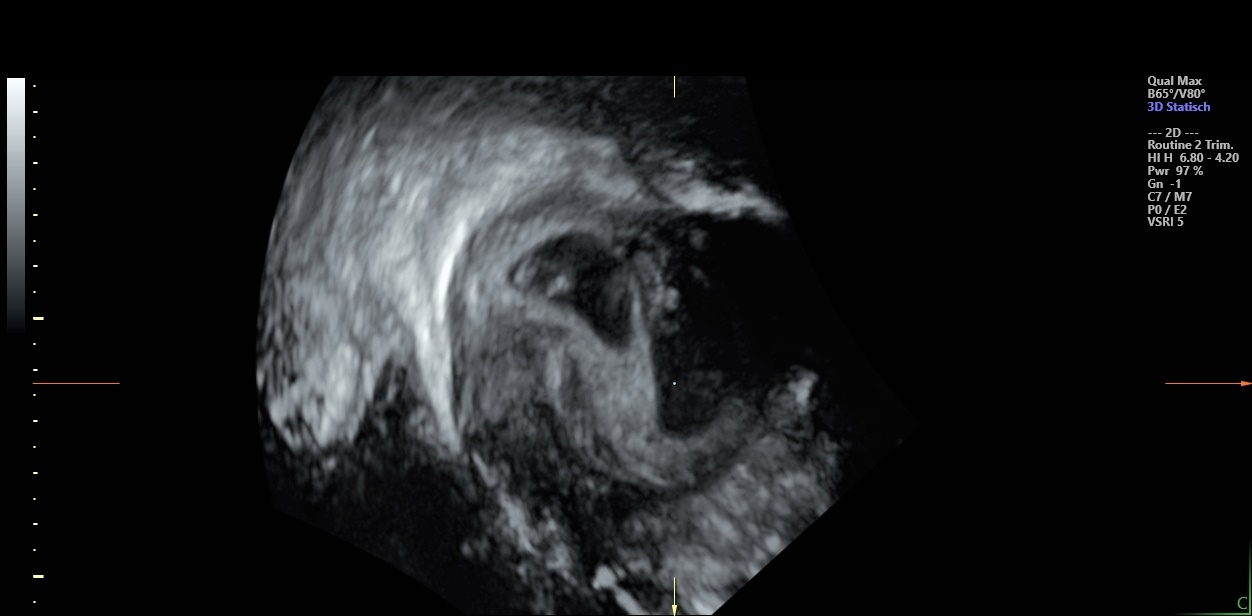

Supplement: Supplementary file 1 — Supplementary Material 1 [file 414_2024_3245_MOESM1_ESM.jpg]
